# Domestic Summary

**Published:** 1839-07-13

**Authors:** 


### DOMESTIC SUMMARY.

*Jefferson Medical College.*—Drs. Pancoast and R. M. Huston have been elected to the Faculty of Jefferson Medical College, to fill the chairs lately occupied by Drs. George M'Clellan and Colhoun.

*Case of abstraction of the Uterus, after delivery.*—At a recent criminal trial in New York, Septimus Hunter was convicted of manslaughter in the fourth degree, for having caused the death of a female patient, by the abstraction of the uterus, after delivery. He was sentenced to a year's imprisonment in the penitentiary. The following report of the case has been furnished to the New York Medical Journal, by Dr. J. H. Griscom:

On the 7th of April, 1839, at the request of Ira B. Wheeler, Esq., coroner, I examined the body of Mrs. Cozins, the wife of a respectable mechanic, No. 328 Madison street, at the time absent from the city. I was assisted in the examination by Dr. S. C. Ellis, in the presence of Drs. Nichols, Lobstein and Walters. Before the examination, we obtained the following history:—Mrs. C. was delivered of a healthy, living child, about one A. M., without any other assistance than her sister and a female friend, both married, and the former a mother. The cord was tied and cut *secundem artem*; but the placenta was retained beyond the usual time. Three hours having elapsed without its disengagement, the sister went for a physician and obtained the services of Septimus Hunter, who represented himself to be a physician, but was at the time a clerk in a drug store. Upon his arrival, he immediately addressed himself to the task of removing the placenta, the successive stages of which operation will be mentioned presently.

We were shown, prior to the dissection, a mass of fleshy substance in a washbowl, which I at once recognised as a uterus; also, in another vessel, the placenta was shown us, which was entire, but without a vestige of the umbilical cord attached to it. The latter was subsequently discovered in a pail of dirty water.

On stripping the body, the abdomen was found very sunken. The usual incisions were made, and the following uncommon appearances were presented: 1st. A total absence of the uterus. 2d. The broad ligaments much torn and ragged, and partly deficient. One fallopian tube was absent, but both ovaria remained *in situ*. 3d. The upper extremity of the vagina was open and free, so that the hand introduced from without would pass directly into the cavity of the abdomen, and the intestines could be touched. The intestines were high up as left by the contracting uterus. 4th. A considerable quantity of extravasated blood was seen on each side near the ovaria, forming spots of ecchymosis beneath the membranes. No effused blood was seen, however, within the abdomen, except this. 5th. A laceration of the vagina, about an inch and a half in length, a short distance from its superior extremity.

By reverting to the uterus, we found the deficient parts attached to it, viz: one fallopian tube, entire; a portion of the broad ligaments, and about an inch of the upper end of the vagina, which had been divided by an even circle, though manifestly without the aid of any cutting instrument. The external surface of the uterus was about half denuded of its peritoneal coat, leaving the muscular fibres entirely bare. Its internal surface was smooth, and the part where the placenta had been attached very apparent, presenting a slight brown colour. The whole organ was about the size of a child's head at birth. Large quanties of coagula were about the body; the bedding was thoroughly soaked with blood, and a large puddle of it, of a bright red colour, covered the floor beneath the bed.

The examination of an intelligent female witness before the coroner's jury, developed the following facts:—Immediately after the *quasi* doctor arrived, he took hold of the cord, and making strong traction upon it, he completely inverted the uterus, the placenta still adhering; pulling still harder, he severed the cord from its attachment and gave it to the witness. He then took hold of the placenta, removed it, and laid it aside, saying there was more to come away still. He then grasped the uterus of the unfortunate patient, and by dint of "excessive" pulling, after about three quarters of an hour, (during which period he relaxed his efforts occasionally to rest and remove his coat, the miserable patient constantly uttering the most piercing and heart-rending cries, such as "you are tearing my heart out, &c.,) he succeeded in dragging the uterus from its attachments, and separated it from the body, holding it in his hands, and exhibiting it as a proof of his prowess and skill, saying that "he never had met with such an extraordinary case before." When asked what it was, he replied, "either a polypus or a false conception." During this brutal operation, the groans of the suffering woman were at first strong and loud; these, together with the force which the man was seen to use, excited the alarm of the attendants, who urged him to desist and allow other medical advice to be called; but with incredible hardihood he persevered, insisting that all was right, that she must endeavour to be patient, and that *he would be responsible for her life.* Towards the close of the performance, her cries became more and more faint, and at length entirely ceased. He thought she was endeavouring to support the pain with patience, and encouraged her in so doing by words. When he turned to look after her, and to feel her pulse, he found that she was dead.

It is due to the profession to say, that the performer of this horrible tragedy is not, *de jure*, a member of the profession, though he asserts that he has a *recommendation* from three surgeons of the British Navy, of his medical proficiency, and that he has had a large amount (three hundred cases) of obstetric practice. He appears to be about thirty-two or thirty-three years of age, and has been in this country two years.

